# Oral and para-oral lymphomas: a 10-year multi-center retrospective study in Egypt with time series analysis and forecasting to 2030

**DOI:** 10.1186/s12903-022-02582-8

**Published:** 2022-12-01

**Authors:** Madiha Ashoub, Mona Wali, Nada Noureldin, Khaled Keraa, Eman El Desouky, Marwa Mokbel ElShafei

**Affiliations:** 1 Oral Pathology Department, Faculty of Oral and Dental Medicine, Misr International University, Cairo, Egypt; 2 Oral and Maxillofacial Pathology Department, Faculty of Dentistry, Cairo University, Cairo, Egypt; 3 Biostatistician, Faculty of Oral and Dental Medicine, Misr International University, Cairo, Egypt; 4 Epidemiology and Biostatistics Department, National Cancer Institute, Cairo University, Cairo, Egypt

**Keywords:** Lymphoma, Oral cavity, Oropharynx, Salivary glands, Epidemiology, Retrospective, Prevalence, Egypt

## Abstract

**Background:**

Little is known about the epidemiology of lymphomas occurring in oral and para-oral sites, especially in developing countries such as Egypt. Hence, the aim of this study was to describe the frequency and time trends of oral and para-oral lymphomas in Cairo governorate from 2010 to 2019, with forecasting to 2030, and to examine relations between age, gender, site and type of lymphoma.

**Methods:**

Histopathological reports of patients diagnosed with oral and para-oral lymphomas from 2010 to 2019 were retrospectively retrieved from archives of six different centers in Cairo governorate. Data regarding age, gender and site was collected and associations between types of lymphoma and these variables were detected using appropriate statistical methods. The significance level was set at *p* ≤ 0.05. Time series analysis was used to determine the trend of lymphoma frequency within 10 years of the study and to predict frequency until 2030.

**Results:**

Lymphomas constituted 2.86% of oral and para-oral lesions. Non-Hodgkin lymphoma was more common than Hodgkin lymphoma. Patients with non-Hodgkin lymphoma showed a higher median age than patients with Hodgkin lymphoma (*p* = 0.001). Non-Hodgkin lymphoma was more prone to occur intra-orally (*p* = 0.014). No statistical significance was observed in gender distribution between Hodgkin and non-Hodgkin lymphoma. Even though no specific time trend was observed from 2010 to 2019, forecasting for frequency of lymphomas through 10 years (2020 to 2030) showed a predicted increase.

**Conclusions:**

The findings of this study were consistent with majority of other studies held in various geographic regions. The study revealed that frequency of oral and para-oral lymphomas in Cairo governorate is expected to rise; hence, oral pathologists should be more clinically suspicious and expect to encounter these lesions more in their practice within the upcoming years.

## Background

Lymphoma is a group of various malignant neoplasms, caused by aberrant proliferation of lymphoid cells or their precursors. It is considered the third most common malignancy worldwide [1]. The incidence of lymphomas has witnessed a substantial increase, including lymphomas occurring in the head and neck region [2]. Compared to other oral and para-oral malignancies, lymphoma comes in third place in terms of frequency of occurrence, following squamous cell carcinoma and salivary gland malignancy [3]. Based on statistics by the World Health Organization (WHO), lymphoma accounts for 3.5% of oral cavity malignancies and 1.7–6% of salivary gland tumors [4].

Despite the rare occurrence of lymphoma in the oral and para-oral region, it is very important for clinicians, oral surgeons and oral pathologists to be aware of its clinicopathological and epidemiological features as well as its distribution among different populations [1, 5]. This is because oral lymphoma may clinically and radiographically resemble other oral lesions such as odontogenic infections or other oral malignancies [6]. Thus, lack of awareness of epidemiological characteristics of oral and para-oral lymphoma can often lead to low index of clinical suspicion which may lead to misdiagnosis, improper treatment and worse prognosis [5, 7].

Not only is it important to determine the prevalence of oral and para-oral lymphomas, but it is also relevant to identify the different types of lymphoma present in these sites. The most recent WHO classification of tumors of hematopoietic and lymphoid tissues continues to divide lymphomas into two main groups: Hodgkin lymphoma (HL) and non-Hodgkin lymphoma (NHL). Even though these two main types of lymphoma can be further subdivided into over 70 different histological subtypes, only some of these subtypes have been reported to occur in oral and para-oral sites [4, 8, 9]. It is important to identify the distinct epidemiological and clinicopathological characteristics of HL and NHL separately as they have been reported to exhibit different behaviors, etiologic factors as well as site, age and gender predilections [10, 11].

Epidemiological studies have shown that there are considerable differences in prevalence of lymphomas among different geographic regions of the world [12]. When compared to other Middle Eastern and Northern African countries for instance, Egypt was reported to have an exceptionally higher frequency of lymphomas [13]. In North Africa, 11,873 cases of lymphomas were prevalent in 2020, 48.9% of which were in Egypt. Besides, NHL was considered the 4^th^ most common cancer in Egypt, accounting for 5.4% of total new malignancies in the year 2020 [14].

Despite the existence of epidemiological data that reflects the frequency of occurrence of lymphomas in general, little is known about the epidemiology of lymphomas occurring specifically in oral and para-oral sites. This owes to the generalization of the data recorded in population-based cancer registries, which collectively describe the incidence of lymphomas without further specification of their distribution among different anatomic sites [15, 16].

In an attempt to fill this research gap, individual countries have exerted efforts towards understanding the epidemiological and clinicopathological features of oral and para-oral lymphomas. These efforts, however, have been mainly restricted to single or multi-center retrospective studies conducted in individual cities. Unfortunately, some parts of the world still lack these epidemiological studies that describe the prevalence of oral and para-oral lymphomas, even on a small institution-based scale. This is especially seen in developing countries, such as those in Africa, where very few of these studies have been reported in literature [17, 18].

As for Egypt, the literature shows how no previous attempts have been made to study and compare the epidemiological characteristics and distribution of various types of oral and para-oral lymphomas. Hence, the main aim of this study was to describe the frequency of different types of oral and para-oral lymphomas in educational hospitals and institutions in Cairo governorate, comparing between HL and NHL based on age, gender and site. Besides, the study intended to observe the trend of lymphoma frequency over the 10 years of the study and accordingly predict the trend within the following 10 years (up till 2030).

## Methods

The study conducted was a multi-centric, observational, retrospective study of patients’ histopathological records. Histopathological reports of patients diagnosed with oral and para-oral lymphomas within the 10-year period from 2010 to 2019 were retrospectively retrieved from archives of the following educational hospitals and institutions in Cairo governorate: Cairo University, Faculty of Dentistry, Oral and Maxillofacial Pathology Department; Cairo University, Faculty of Medicine, General Pathology Department; Ain Shams University, Faculty of Dentistry, Oral Pathology Department; Al-Azhar University, El-Sayed Galal Hospital; Ahmed Maher Teaching Hospital and National Cancer Institute.

Inclusion criteria involved cases diagnosed within the 10 years (2010–2019); all age groups and both genders; lymphomas occurring in oral cavity, oropharynx and salivary glands and all histological subtypes of Hodgkin and non-Hodgkin lymphoma. On the other hand, all other types of hematolymphoid tumours were excluded, as well as cases of lymphoma diagnosed without histopathological proof.

The histopathological reports of patients that met the inclusion and exclusion criteria were either checked manually or were electronically retrieved from digital databases using appropriate search terms. Data regarding age, gender, site and diagnosis was collected and recorded from the histopathological reports. The diagnosis obtained from the included cases was either based on histological description written in the reports or description of both histological and immunohistochemical analyses.

Review of the diagnosis written in every histopathological report was carried out by the same investigator, to unify the data abstraction and analysis method from several sources. Based on the WHO classification of tumors of hematopoietic and lymphoid tissues, lymphoma was divided into Hodgkin lymphoma (HL) or non-Hodgkin lymphoma (NHL). The histological type of lymphoma was correlated with age, gender and site of affection.

### Ethics approval

This study was approved by The Research Ethics Committee at Faculty of Dentistry, Cairo University (No. 16 12 19). Patients’ names included in the histopathological reports were kept confidential and were not used in this study.

### Statistical analysis

The data gathered was entered into Microsoft Excel spreadsheets, and transferred to IBM SPSS Statistics for Windows, Version 23.0. Armonk, NY: IBM Corp. statistical software program for analysis. Qualitative data were presented as frequencies and percentages. Quantitative data were presented as mean, standard deviation (SD), median, range and 95% confidence interval (95% CI). Chi-square test or Fisher’s Exact test was used for comparisons regarding qualitative variables. Quantitative data (age data) were explored for normality by checking the distribution of data and using tests of normality (Kolmogorov–Smirnov and Shapiro–Wilk tests). Age data showed non-normal (non-parametric) distribution; so Mann–Whitney U test was used to compare between ages of patients with NHL and HL. Binary logistic regression analysis was used to determine significant predictors of NHL and HL. Model fit was tested using Chi-square test and the model was fit to describe the relations between the dependent and independent variables. The regression coefficient (β), standard error (SE), and 95% confidence interval (95% CI) were calculated. Time series analysis was performed to determine the trend of lymphoma frequency within the 10 years of the study (2010–2019) as well as to predict the frequency of lymphoma for a 10-year period after the study (2020–2030). The significance level was set at *p* ≤ 0.05.

## Results

There were 362 cases of oral and para-oral lymphomas out of 12,662 oral and para-oral lesions diagnosed in the study centers in the 10-year period (2010–2019), giving an overall frequency of (2.86%). Non-Hodgkin lymphoma (NHL) was the more common type (*n* = 326, 90.1%), compared to Hodgkin lymphoma (HL) (*n* = 15, 4.1%). 21 lesions (5.8%) were diagnosed as lymphoma without specifying the type (lymphoma, NOS = lymphoma, not otherwise specified).

### Association between age and type of lymphoma

**Fig. 1:**
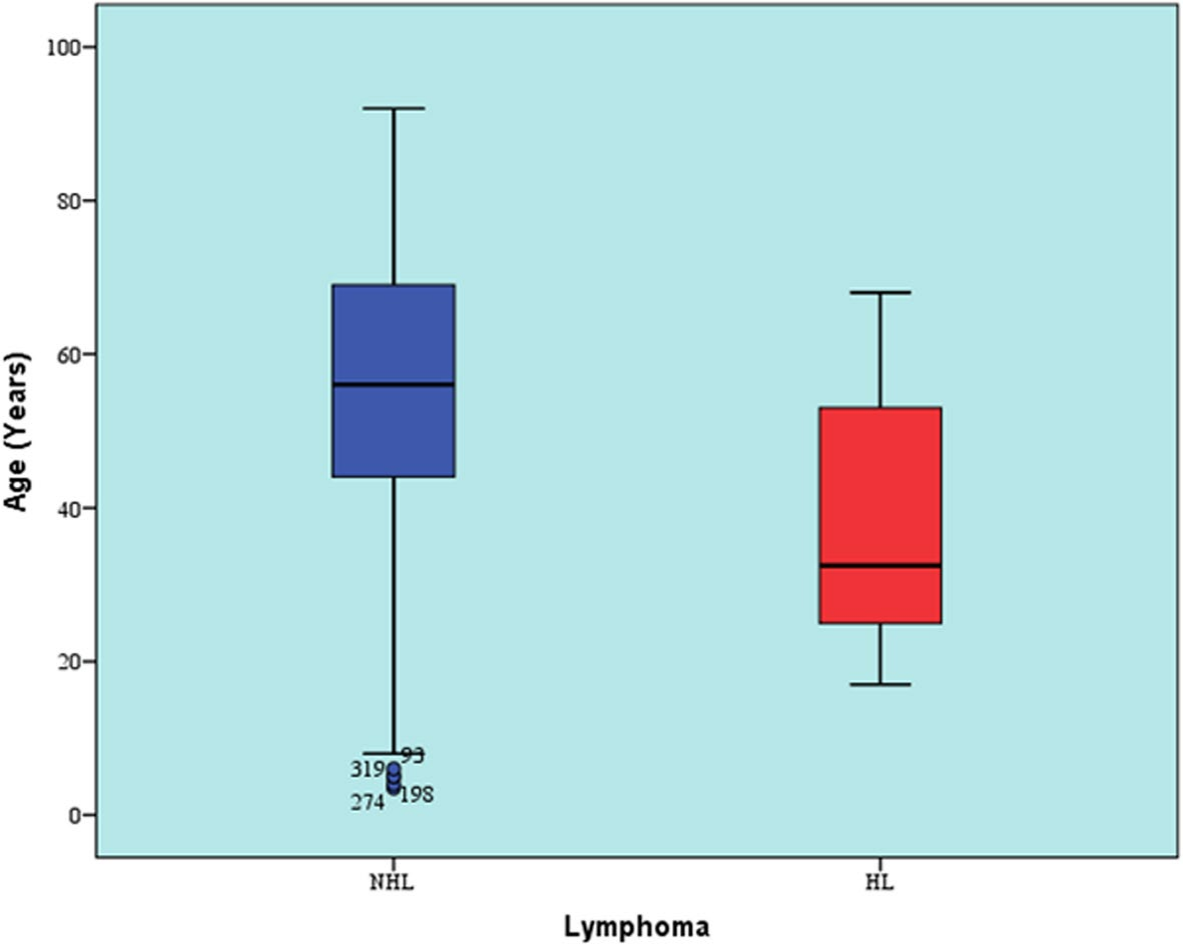
Box plot representing median and range values for ages of patients with HL and NHL (Circles represent outliers)

**Fig. 2:**
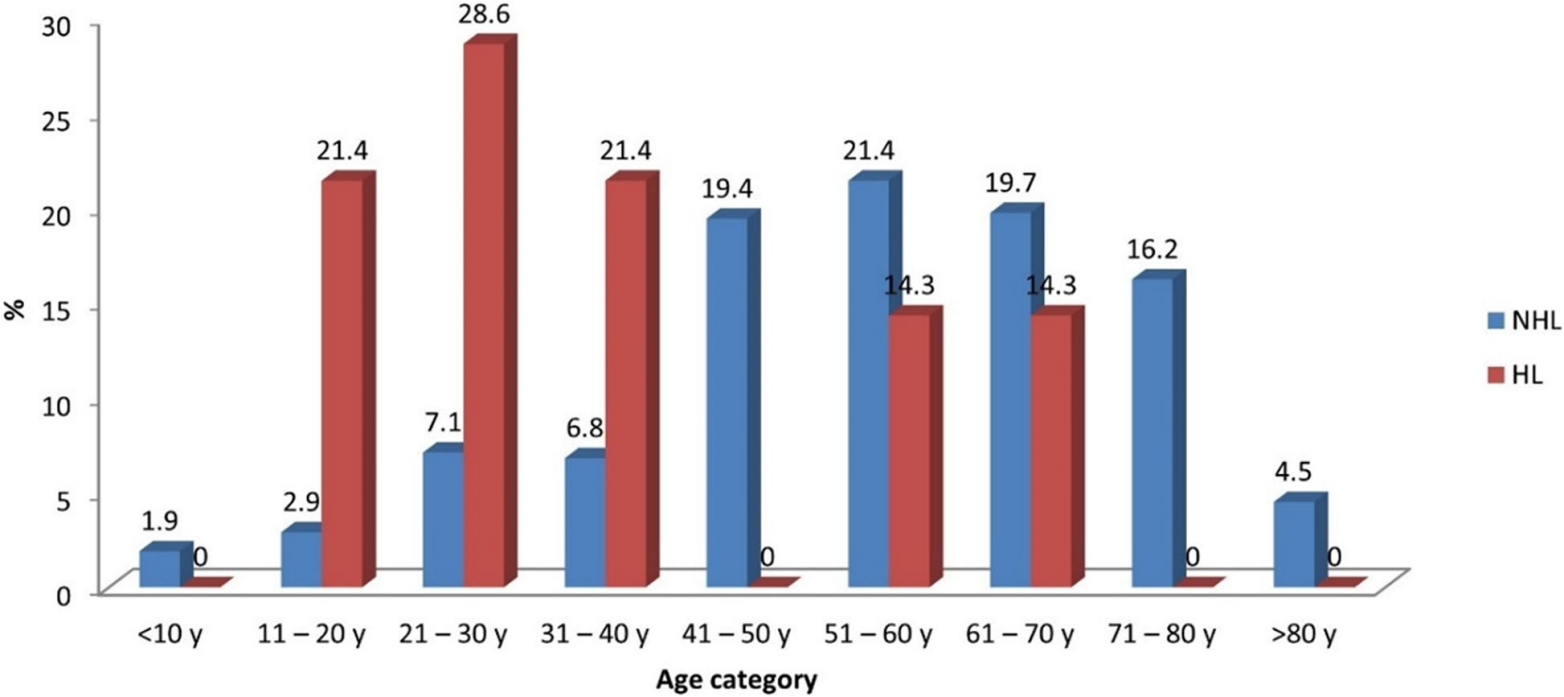
Bar chart representing the association between age categories and type of lymphoma

Patients with NHL showed statistically significant higher median age than patients with HL (*p*-value = 0.001, effect size = 0.397). Comparison of age values between HL and NHL is illustrated in Table 1 and Fig. 1. Furthermore, there was a statistically significant association between age category and type of lymphoma (*p*-value = 0.001, effect size = 0.306), as demonstrated in Table 2 and Fig. 2. Compared to HL, NHL was more prevalent in patients aged less than 10 years old and patients aged more than 40 years old. On the other hand, HL was more prevalent than NHL in patients aged 11 – 40 years old.

**Table 1:** Descriptive statistics and results of Mann–Whitney U test for comparison between age values in patients with NHL and HL

NHL (*n* = 309)^a^		HL (*n* = 14)^a^		*p*-value	*Effect size (d)*
Mean (SD)	Median (Range)	Mean (SD)	Median (Range)		
54.5 (18.3)	56 (3.5–92)	37.2 (16.7)	32.5 (17–68)	0.001*	0.397

^a^17 cases were excluded from NHL and one case was excluded from HL group because age was not specified

^*^Significant at *p* ≤ 0.05

**Table 2:** Descriptive statistics and results of Fisher’s Exact test for the association between age categories and type of lymphoma

Age category	NHL (*n* = 309)^a^		HL (*n* = 14)^a^		*p*-value	*Effect size (v)*
	n	%	n	%		
< 10 y	6	1.9	0	0	0.001*	0.306
11 – 20 y	9	2.9	3	21.4		
21 – 30 y	22	7.1	4	28.6		
31 – 40 y	21	6.8	3	21.4		
41 – 50 y	60	19.4	0	0		
51 – 60 y	66	21.4	2	14.3		
61 – 70 y	61	19.7	2	14.3		
71 – 80 y	50	16.2	0	0		
> 80 y	14	4.5	0	0		

^a^17 cases were excluded from NHL and one case was excluded from HL group because age was not specified

^*^Significant at *p* ≤ 0.05

### Association between gender and type of lymphoma

Specific histologic subtypes of NHL showed different gender distributions, as shown in Table 3. In extra-nodal marginal zone lymphoma of mucosa-associated lymphoid tissue (MALT lymphoma), females were mostly commonly affected (69.6%), while males were affected in (26.1%) of cases. In Burkitt lymphoma, male predominance was found as males constituted (81.8%) of cases, while females were affected in (18.2%) of cases.

**Table 3:** Frequencies (n) and percentages (%) for gender distribution among histologic subtypes of NHL (*n* = 279)

Histologic subtypes of NHL	Male		Female		Unspecified	
	n	%	n	%	n	%
DLBCL (*n* = 191)	89	46.6	94	49.2	8	4.2
MALT lymphoma (*n* = 46)	12	26.1	32	69.6	2	4.3
Burkitt lymphoma (*n* = 11)	9	81.8	2	18.2	-	-
Follicular lymphoma (*n* = 7)	4	57.1	3	42.9	-	-
CLL/SLL (*n* = 7)	4	57.1	3	42.9	-	-
Anaplastic large T-cell lymphoma (*n* = 4)	3	75	1	25	-	-
T-cell/histiocyte rich B-cell lymphoma (*n* = 3)	1	33.3	2	66.7	-	-
Mantle cell lymphoma (*n* = 2)	-	-	2	100	-	-
B-lymphoblastic lymphoma (*n* = 2)	-	-	2	100	-	-
Extra-nodal NK/T-cell lymphoma (*n* = 2)	1	50	1	50	-	-
Peripheral T-cell lymphoma (*n* = 2)	2	100	-	-	-	-
Plasmablastic lymphoma (*n* = 1)	1	100	-	-	-	-
T-lymphoblastic lymphoma (*n* = 1)	-	-	1	100	-	-

*DLBCL* Diffuse large B-cell lymphoma, *CLL/SLL* Chronic lymphocytic leukemia/small lymphocytic lymphoma

**Fig. 3:**
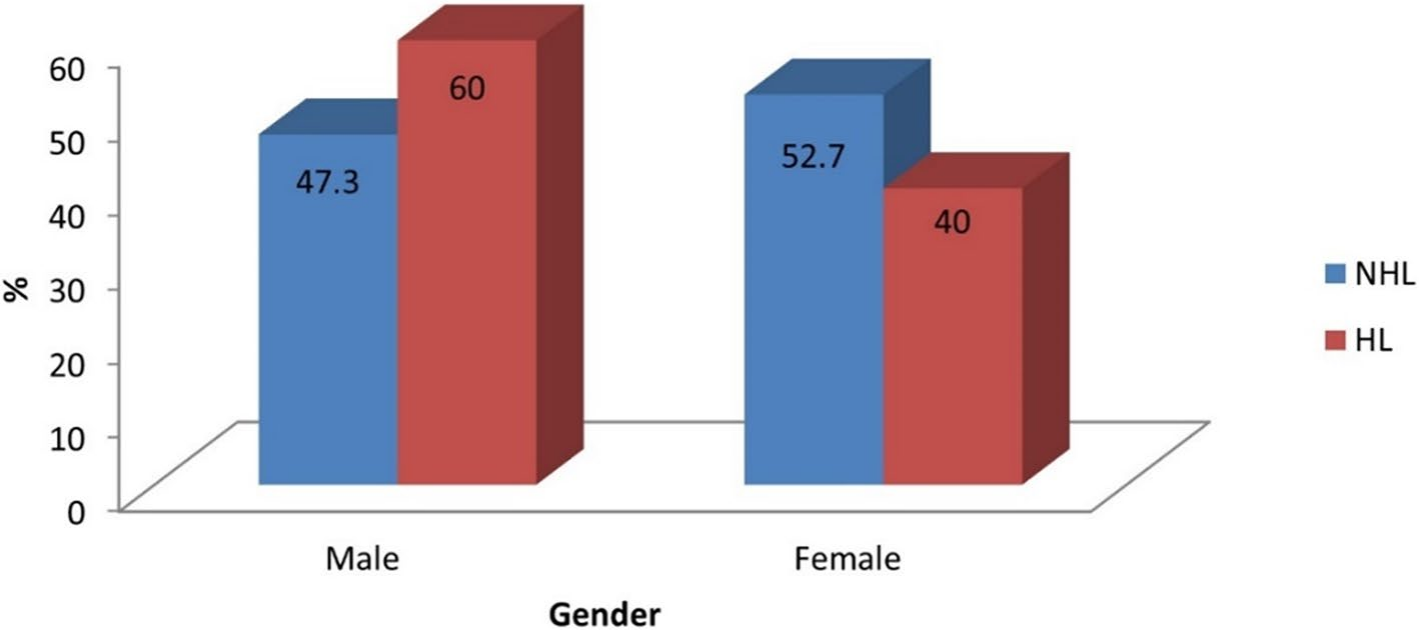
Bar chart representing the association between gender and HL and NHL

However, there was no statistically significant association between gender and HL and NHL (*p*-value = 0.336, effect size = 1.633). Males are 1.633 folds prone to HL than females. Table 4 and Fig. 3 illustrate the association between gender and HL and NHL.

**Table 4:** Descriptive statistics and results of Chi-square test for the association between gender and HL and NHL

Gender	NHL (*n* = 315)^a^		HL (*n* = 15)		*p*-value	*Effect size (OR)*
	n	%	n	%		
Male	149	47.3	9	60	0.336	1.633
Female	166	52.7	6	40		

^a^11 cases were excluded from NHL because gender was not specified

^*^Significant at *p* ≤ 0.05

### Association between site and type of lymphoma

**Fig. 4:**
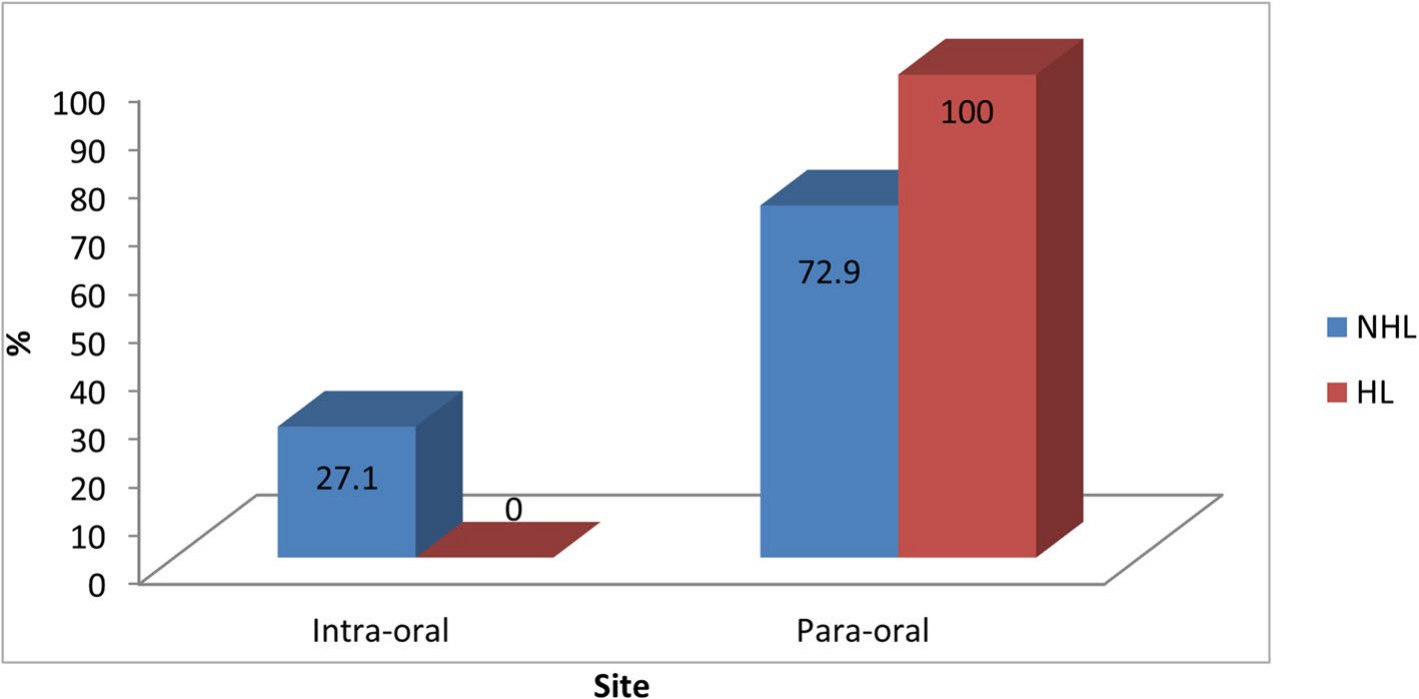
Bar chart representing the association between site and type of lymphoma

There was a statistically significant association between site and type of lymphoma, as shown in Table 5 and Fig. 4 (*p*-value = 0.014, effect size = 1.063). NHL is 1.063 folds more prone to occur intra-orally compared to HL.

**Table 5:** Descriptive statistics and results of Fisher’s Exact test for the association between site and type of lymphoma

Site	NHL (*n* = 325)^a^		HL (*n* = 15)		*p*-value	*Effect size (OR)*
	n	%	n	%		
Intra-oral	88	27.1	0	0	0.014*	1.063
Para-oral	237	72.9	15	100		

^a^One case was excluded from NHL group because site was not specified

^*^Significant at *p* ≤ 0.05

**Fig. 5:**
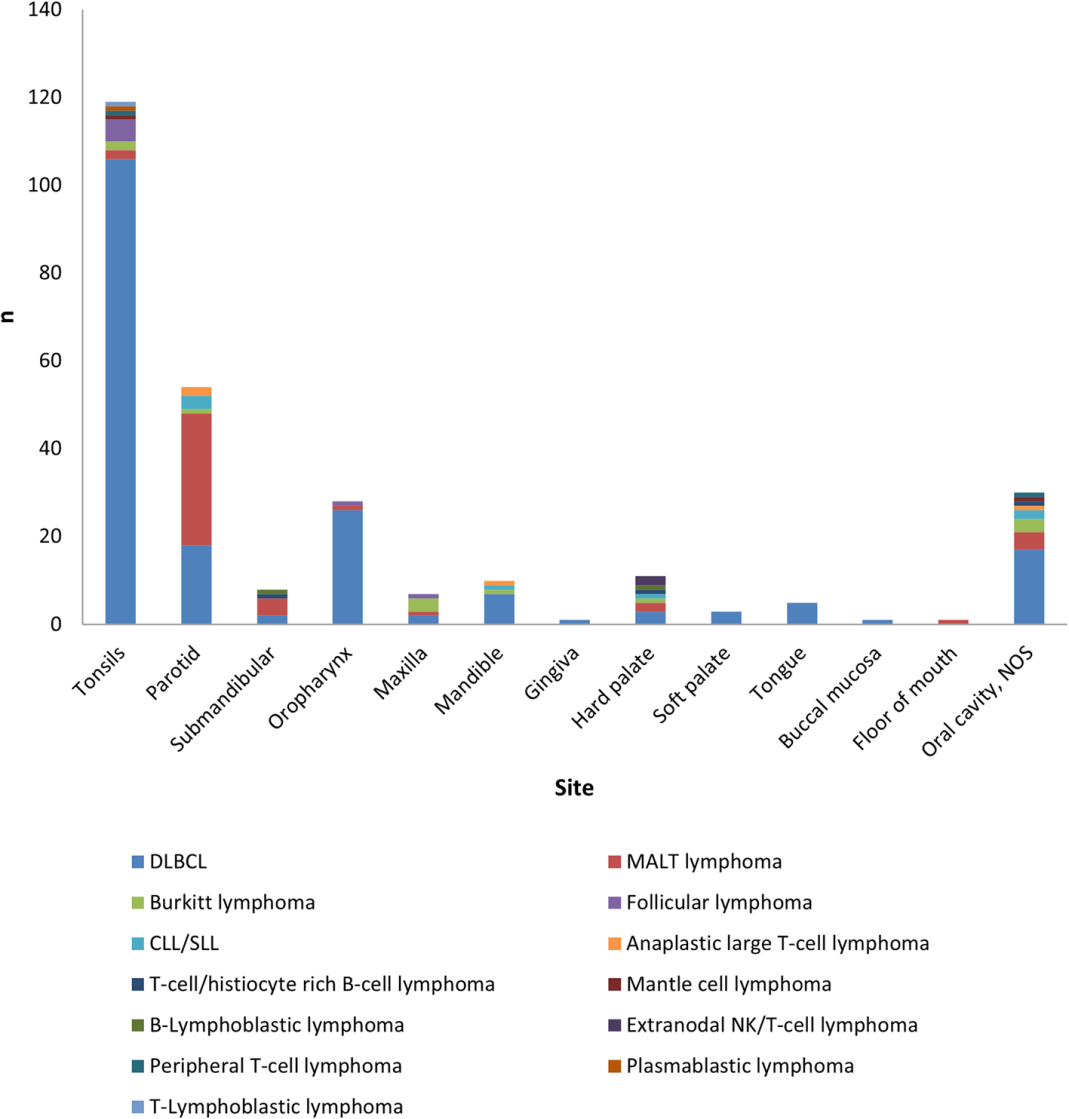
Bar chart showing distribution of different histological subtypes of NHL among oral and para-oral sites

Figure 5 shows the frequency of distribution of each histologic subtype of NHL among different oral and para-oral sites. Diffuse large B-cell lymphoma (DLBCL) was the most common histologic subtype to occur in tonsils, while MALT lymphoma was the most common subtype in parotid and submandibular glands.

### Regression analysis

Binary logistic regression model was constructed using NHL, HL (binary outcome) as the dependent variable while age and gender were the independent variables. Model fitting was tested by the statistically significant Chi-square test (Chi-square = 10.768, *p*-value = 0.005). Results of the regression model, presented in Table 6, showed that age is a statistically significant predictor for prevalence of lymphoma; older people are more prone to NHL than younger ones (Regression coefficient = -0.054, OR = 0.956, *p*-value = 0.002). Gender was not found to be a significant predictor of lymphoma.

**Table 6:** Results of binary logistic regression analysis model showing predictors of NHL and HL

Variable	Regression coefficient (β)	Standard Error (SE)	*p*-value	OR	95% CI for OR
Age	-0.045	0.014	0.002*	0.956	0.929 – 0.983
Gender (Reference category: Male)	-0.094	0.575	0.871	0.911	0.295 – 2.81

^*^Significant at *p* ≤ 0.05

### Time series analysis

**Fig. 6:**
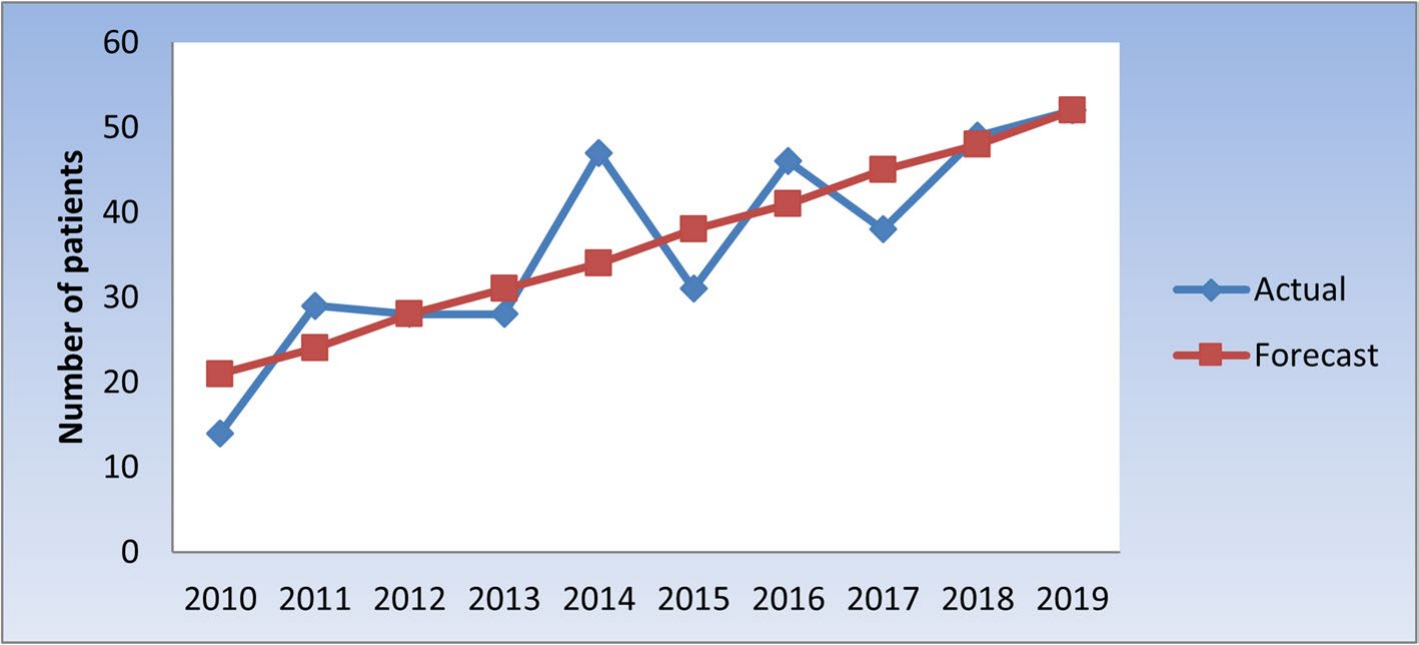
Actual and predicted (forecast) number of patients with oral and para-oral lymphomas through the years 2010 to 2019

**Fig. 7:**
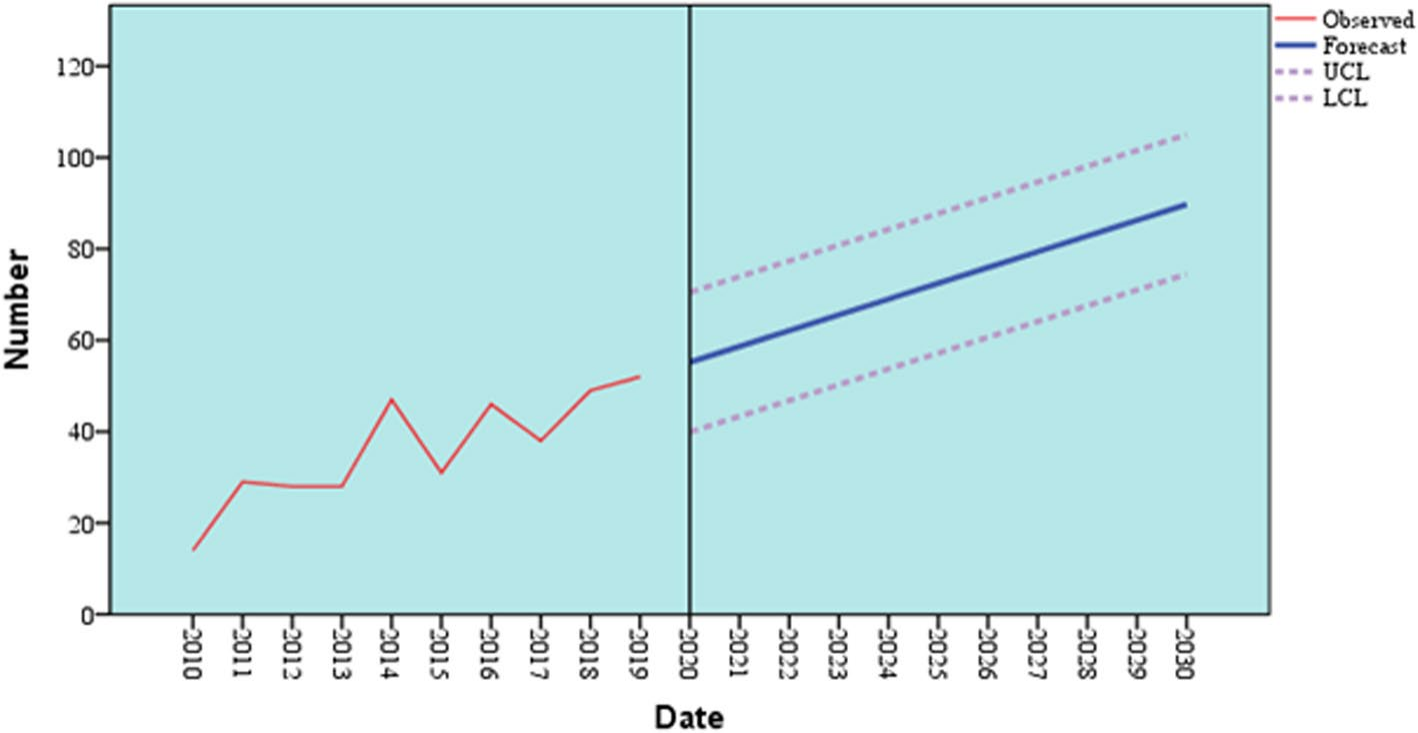
Predicted (forecast) number of patients with oral and para-oral lymphomas through the years 2020 to 2030 and its 95% Confidence Interval (UCL: Upper Confidence Limit, LCL: Lower Confidence Limit)

Table 7 and Fig. 6 show the actual and predicted number of patients with oral and para-oral lymphomas through the period (2010 – 2019). Time series analysis showed no trend of increase or decrease in number of patients with oral and para-oral lymphomas from 2010 to 2019. Forecasting (prediction) for prevalence of oral and para-oral lymphomas through 10 years (2020 to 2030) showed a predicted increase, as demonstrated in Table 8 and Fig. 7.

**Table 7:** Actual and predicted number of patients with oral and para-oral lymphomas through the period (2010 – 2019)

Year	Actual (observed) number	Predicted (forecast) number
2010	14	21
2011	29	24
2012	28	28
2013	28	31
2014	47	34
2015	31	38
2016	46	41
2017	38	45
2018	49	48
2019	52	52

**Table 8:** Predicted number of patients with oral and para-oral lymphomas through the period (2020 – 2030)

Year	Predicted (forecast) number	95% Confidence Interval
2020	55	40 – 70
2021	59	43 – 74
2022	62	47 – 77
2023	66	50 – 81
2024	69	54 – 84
2025	72	57 – 88
2026	76	61 – 91
2027	79	64 – 95
2028	83	68 – 98
2029	86	71 – 102
2030	90	74 – 105

## Discussion

Epidemiology and pathology are two independent yet interrelated fields of science that form two indispensable pillars of medical research. The integration of epidemiology in pathology benefits pathologists in more than one way. First and foremost, epidemiological prevalence studies measure the frequency of disease occurrence in specific populations, thus reflecting the burden of disease in each population. This awareness increases pathologists’ index of suspicion to various diseases, which in turn helps them in formulating accurate differential diagnoses [19, 20]. Acknowledging the central dogma of science, which revolves around the fact that diseases do not haphazardly occur, one of the main goals of epidemiology is to determine the causes and possible risk factors of diseases. In addition, comparison between results of prevalence studies held in different geographic regions can result in the identification of possible environmental factors that might influence the occurrence of disease in certain regions [19–21].

The current study described the frequency and annual trends of oral and para-oral lymphomas diagnosed within a 10-year period (2010 to 2019) in Cairo governorate’s educational hospitals and institutions. Relations between age, gender, site and type of lymphoma were also analyzed. The main aim of the study was to contribute in filling the research gap that exists as epidemiology of oral and para-oral lymphomas has not yet been reported in Egypt, while also predicting the frequency of lymphomas within the 10 years following the study (until 2030).

Results revealed that in the 10-year period from 2010 till 2019, a total of 12,662 lesions occurring in oral and para-oral sites were archived in the pathology units of six of Cairo governorate’s educational hospitals and institutions. Out of these 12,662 cases, 362 (2.86%) were histologically diagnosed as lymphomas. Other than these 362 oral and para-oral lymphoma cases, 38 cases were recorded as suggestive for lymphoma. Accordingly, these cases were excluded from the study due to the lack of enough histological or immunohistochemical evidence to support a definitive final diagnosis.

In their epidemiological study that was based in South Africa, Alli and Meer found that head and neck lymphomas constituted 2.2% of the total number of cases which, albeit lower than, is still close to the percentage recorded in our study [17]. On the other hand, the frequency of oral and para-oral lymphomas in our study is much higher than those recorded in the two studies performed by Keszler et al. in Argentina and Kusuke et al. in Brazil, which were 0.2% and 0.1% respectively [7, 22].

Even though comparing results between different studies is highly encouraged, it is not always feasible to compare percentages that reflect frequency of disease occurrence. This is because these percentages differ depending on each study’s inclusion criteria. While the current study calculated the frequency of all types of lymphomas occurring in oral cavity, oropharynx and salivary glands in relation to the total number of lesions diagnosed in these sites, Keszler et al. and Kusuke et al. specifically measured the proportion of only NHL occurring in the oral cavity [7, 22]. These differences could explain why the percentage of lymphomas in our study is greater as we included all types of HL and NHL and did not restrict the site of occurrence to merely the oral cavity.

The reason behind including other sites to the oral cavity in our study- specifically the oropharynx and salivary glands- is because these sites, which we referred to as para-oral sites, have been reported to harbor different types of lymphomas. The Waldeyer tonsillar ring, for instance, is considered the most common site for extra-nodal head and neck lymphoma; the palatine tonsils, which are located in the oropharynx, are mostly involved [4, 23]. Concerning salivary glands, both the gland parenchyma and intra-glandular lymph nodes can be affected by lymphoma. Accordingly, salivary glands can be affected by either extra-nodal or nodal lymphomas respectively [24]. On the other hand, our study did not include lymphomas occurring in the neck lymph nodes because oral pathologists mainly encounter cases related to the oral cavity, oropharynx and salivary glands [25], so these are the only three major relevant sites we had decided to include in our study.

The nature of the total lesions against which the proportion of lymphoma cases was calculated also varied between studies, which presented as another challenge when comparing results of different studies. In our investigation, the total number of lesions (*n* = 12,662) involved any type of lesion that occurred in oral or para-oral sites, regardless of nature or behavior. On the other hand, there were studies that calculated the frequency of head and neck lymphomas relative to the total number of head and neck malignancies, excluding any benign or reactive lesions from the total count [26–28]. Moreover, Guevara-Canales et al. calculated the proportion of oral and para-oral lymphomas in relation to the total number of lymphomas found in the human body [29].

In the current study, no specific trend for increase or decrease in the frequency of oral and para-oral lymphomas was observed over time. The lowest percentage of lymphomas was seen in 2010 while the highest percentage was in 2014. Since the data collected in the current study did not include information regarding medical history, occupation or presence of certain etiologic factors, any interpretations for the variation in lymphoma incidence throughout the years would merely be assumptions that would require further analytical studies to prove.

Observing the annual frequency of oral and para-oral lymphomas from 2010 to 2019 also enabled us to predict their occurrence in the following 10 years, 2020 to 2030. The time series analysis performed showed that the frequency of oral and para-oral lymphomas is expected to show a uniform increase throughout the 10 years (2020–2030). This coincides with the results of the National Cancer Registry Program of Egypt that were published in 2014, where an estimated rise in incidence rates of HL and NHL until the year 2050 was predicted [15]. Even though the present study deals with the frequency of lymphomas specifically occurring in oral and para-oral sites, it could still be correlated to the overall incidence of lymphomas occurring in different anatomic sites.

NHL was the more common type (90.1%), compared to HL which represented (4.1%) of the study sample. These results coincide with the findings of other studies conducted on oral and para-oral lymphomas in various parts of Canada, UK, Brazil, Iran and France. These studies even reported slightly higher proportions of NHL than our study, ranging from 94.5 to 100% of all included cases [26, 27, 29–32]. The higher frequency of NHL in oral and para-oral sites can be explained by the fact that NHL can frequently occur in both nodal and extra-nodal sites, unlike HL which has a propensity for nodal sites.

Studies that included various nodal sites of the head and neck also reported higher proportion of NHL in relation to HL. However, the percentage of HL cases in these studies was higher than the percentage reported in our study. For example, in the study conducted by Urquhart and Berg in USA, 24.4% of head and neck lymphomas were of HL type [33]. In addition, another study that was carried out by Shamloo et al. in Iran reported 39% of head and neck lymphomas to be HL [34]. The higher percentages of HL in these studies compared to our study (4.1%) can be explained by the inclusion of the neck lymph nodes in these studies; HL has a higher probability of occurring in nodal sites than in extra-nodal sites.

Even though our study did not include lymphomas occurring in neck lymph nodes, Hodgkin lymphoma was still detected as it represented 4.1% of the study sample. Despite being mainly a nodal disease, the literature shows how 1–4% of Hodgkin lymphomas occur in extra-nodal areas [35, 36]. Although rare, HL has been documented to occur in oral and para-oral sites such as in salivary gland lymph nodes, or in extra-nodal sites such as tonsils, oropharynx and palate [37, 38].

An association between age and type of lymphoma was found in the present study. Patients with NHL showed a statistically significant higher median age than patients with HL. This is in accordance with the results of other studies conducted in USA, Iran and South Africa [17, 28, 33, 34]. Comparing the age range between NHL and HL, it was found that NHL encompassed a wider age range (3.5–92 years) than HL (17–68 years). This could be explained by the fact that NHL includes a wide variety of histological subtypes with different age predilections [39]. Burkitt lymphoma, for instance, is known to be predominant in infants and young adults [40]; this was also evident in our study as the youngest age (3.5 years) in the study sample was of a case diagnosed as Burkitt lymphoma. On the other hand, other types of lymphoma, such as DLBCL, are more common in adults. Besides, the frequency of DLBCL actually increases with increasing age [41, 42]. Thus, the presence of different subtypes of NHL in the study sample explains the wide age range observed in this study.

Age was also found to be a statistically significant predictor for occurrence of oral and para-oral lymphomas; older people were proven to be more prone to NHL than younger ones. Older patients are generally believed to be more susceptible to immune deficiency as a result of organ transplantation or auto-immune diseases. Since immune deficiency has been recognized in literature as a risk factor for NHL, this could explain why older people are at a higher risk for developing NHL. Besides, the cumulative effect of prolonged exposure to carcinogenic agents could increase the risk of lymphomagenesis in the elderly.

Regarding gender, statistical analysis showed no significant difference between male and female affection for both HL and NHL. This is in agreement with results of similar studies conducted in USA, South Africa, Germany and Brazil that all reported insignificant gender predilection in cases of oral and para-oral lymphomas [1, 17, 33, 43].

However, specific histologic subtypes of NHL presented with distinctive gender distributions in our study. In MALT lymphoma for instance, 69.6% of cases were females. Similarly, a study conducted by Iguchi et al. in Japan also reported female predominance in MALT lymphoma occurring in the head and neck region [44]. MALT lymphoma is generally observed in females more than males, especially when it occurs in the salivary glands [45]. This is because salivary MALT lymphoma is often associated with the presence of Sjogren syndrome, which is mainly a female disease [33, 46].

On the other hand, Burkitt lymphoma showed male predominance in the current study, with 81.8% of cases occurring in males. Similar findings were reported in Iran, Germany, Kenya and Brazil [1, 18, 27, 43]. According to Stefan and Lutchman, even though male predominance is usually observed in Burkitt lymphoma, gender does not affect the survival outcome of the disease [47].

The present study revealed a statistically significant association between site and type of lymphoma. NHL was found to be 1.063 folds more prone to occur intra-orally, compared to HL. Since NHL has a greater propensity to occur in extra-nodal sites than HL, this could explain the higher frequency of NHL in intra-oral sites. This knowledge could serve very useful for oral surgeons and pathologists to formulate a differential diagnosis when they encounter an intra-oral lesion that is suspicious for lymphoma.

In addition, certain anatomic sites presented with increased predilection for specific histologic subtypes of NHL. In tonsils, the most common histologic subtype of lymphoma observed was DLBCL. On the other hand, MALT lymphoma was the most common type of NHL in both the parotid and submandibular lymph nodes. A study conducted by Hart et al. in UK also reported the same findings regarding predominance of DLBCL in tonsils and MALT lymphoma in salivary glands [32].

MALT lymphoma is usually preceded by a chronic inflammatory process or auto-immune disease that stimulates the proliferation of lymphoid tissue in extra-nodal sites, thus forming what is known as, acquired mucosa-associated lymphoid tissue. The salivary glands are target organs for formation of acquired mucosa-associated lymphoid infiltration as a result of affection by Sjogren syndrome. Accordingly, MALT lymphoma of the head and neck has a predilection for salivary glands.

Even though this research managed to describe the frequency and distribution of different types of oral and para-oral lymphomas in Cairo governorate’s educational hospitals and institutions, several limitations were encountered throughout the data collection and analysis processes. Owing to the retrospective nature of the study, the issue of missing data was inevitably faced. Besides, some lesions were diagnosed as suggestive for lymphoma, without histological or immunohistochemical verification of diagnosis and were therefore excluded from the study.

In many instances, the characterization of lymphoma subtypes requires more advanced diagnostic investigations than the traditional viewing of the microscopic slides stained with Hematoxylin and Eosin. Furthermore, these advanced procedures are not only restricted to immunohistochemical staining of specific markers, but molecular studies have also become a major player in differentiating some aggressive subtypes of lymphomas, such as diffuse large B-cell lymphoma/high grade large B-cell lymphoma with MYC and BCL2 rearrangements, from DLBCL, NOS. It is important to differentiate between these subtypes of lymphomas, since they have different prognoses and thus respond differently to certain treatments [9, 42]. However, these diagnostic tests are highly expensive, and are not readily available in most pathology centers, especially in developing countries such as Egypt.

Although the results of the current study revealed associations between age, gender, site and types of lymphomas, the nature of the study still did not allow for analysis of the effects of possible etiologic factors, such as certain viral infections or auto-immune diseases, on the prevalence of lymphomas. Plasmablastic lymphoma, for instance, is considered the number one type of lymphoma to be related to HIV-associated immunosuppression; HIV positivity has been detected in 92% of intra-oral plasmablastic lymphoma patients [48]. Compared to other geographic regions, the prevalence of HIV infection in Egypt and other Arab Northern African countries is considered among the lowest rates of infection in the world [49]. Nevertheless, a 31% rise in HIV positive cases has been witnessed in Middle Eastern countries since 2001 [50]. Statistics by UNAIDS have shown that in 2021, 30,000 people in Egypt were living with AIDS [51].

Despite the relatively low prevalence of HIV infection in Egypt, it is still important to study the association between HIV and the prevalence of different types of lymphoma, such as plasmablastic lymphoma. This data could prove useful as some studies have suggested that the survival rates of patients with plasmablastic lymphoma differ depending on the HIV status of patients [52]. As the data collected in the current study did not include such information, studying the effects of certain etiologic factors, such as HIV infection, on frequency of different types of oral and para-oral lymphomas would require further analytical studies to prove.

Since the literature lacked studies that observed the time trends and epidemiological characteristics of oral and para-oral lymphomas in Egypt, this study acts as a stepping stone towards understanding the epidemiology of such lesions in the population, while providing predictions for the prevalence of such lesions in the future.

## Conclusions

In light of the results and interpretations presented in the current study, it can be concluded that lymphomas can indeed occur in oral and para-oral sites, as they constituted 2.86% of the oral and para-oral lesions diagnosed. For the most part, the results of the current study compare well with studies conducted in other populations. The study revealed that NHL was the most common type of lymphoma to occur in oral and para-oral sites, which corresponds well to the overall predominance of NHL in all body sites, as reported by the National Cancer Registry Program of Egypt. According to our results, older age was documented as a risk factor for the development of NHL in oral and para-oral sites. Projecting to 2030, frequency of oral and para-oral lymphomas in Cairo governorate is expected to rise, according to the results of this study. Hence, oral pathologists and surgeons should be alarmed about the predicted increase in the burden of oral and para-oral lymphomas in Cairo governorate and expect to encounter them more in their practice within the upcoming years.

## Data Availability

The data that support the findings of this study are available from the corresponding author upon request.

## Abbreviations

WHO: World Health Organization
NHL: Non-Hodgkin lymphoma
HL: Hodgkin lymphoma
MALT lymphoma: Extra-nodal marginal zone lymphoma of mucosa-associated lymphoid tissue
DLBCL: Diffuse large B-cell lymphoma
CLL/SLL: Chronic lymphocytic leukemia/small lymphocytic lymphoma
